# Supplementary material for: Unitary Coupled-Cluster Theory for the Treatment of Molecules in Strong Magnetic Fields
Source: J Chem Theory Comput. 2025 Dec 8;21(24):12634–51. doi: 10.1021/acs.jctc.5c01521 (PMC12746449; doi:10.1021/acs.jctc.5c01521)
Supplement: Supplementary file 1 [file ct5c01521_si_001.pdf]

Unitary Coupled-Cluster theory for the treatment of molecules in strong magnetic fields

Laura Grazioli,[*,†,‡,¶] Marios-Petros Kitsaras,[*,§,‡,¶] and Stella Stopkowicz[*,¶,‖]

†*CERMICS, École nationale des ponts et chaussées, 6 et 8 avenue Blaise Pascal, Cité Descartes, 77455 Marne la Vallée Cedex 2, France*

‡*Department Chemie, Johannes Gutenberg-Universität Mainz, Duesbergweg 10-14, D-55128 Mainz, Germany*

¶*Fachrichtung Chemie, Universität des Saarlandes, Campus B2 2, D-66123 Saarbrücken, Germany*

§*Laboratoire de Chimie et Physique Quantiques - UMR5626, CNRS, Université de Toulouse - Bat. 3R1b4 - 118 route de Narbonne, F-31062, Toulouse, France*

‖*Hylleraas Centre for Quantum Molecular Sciences, Department of Chemistry, University of Oslo, P.O. Box 1033, N-0315 Oslo, Norway*

E-mail: laura.grazioli@enpc.fr; kitsaras@irsamc.ups-tlse.fr; stella.stopkowicz@uni-saarland.de

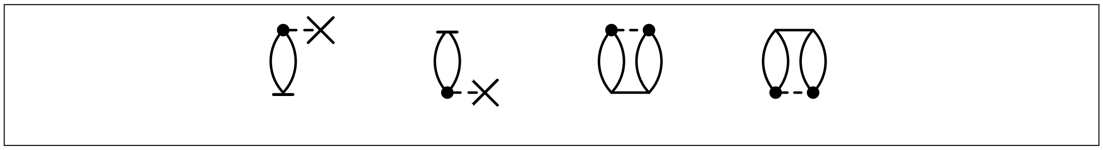

Diagrams S1: Diagrams describing the UCC energy; these are the same both in the UCC2 and UCC3 framework. The continuous lines depict the $\hat{\sigma}$ operator, the dashed line with a cross is the Fock operator, while the dashed line with two vertices symbolizes the operator $\hat{V}$.

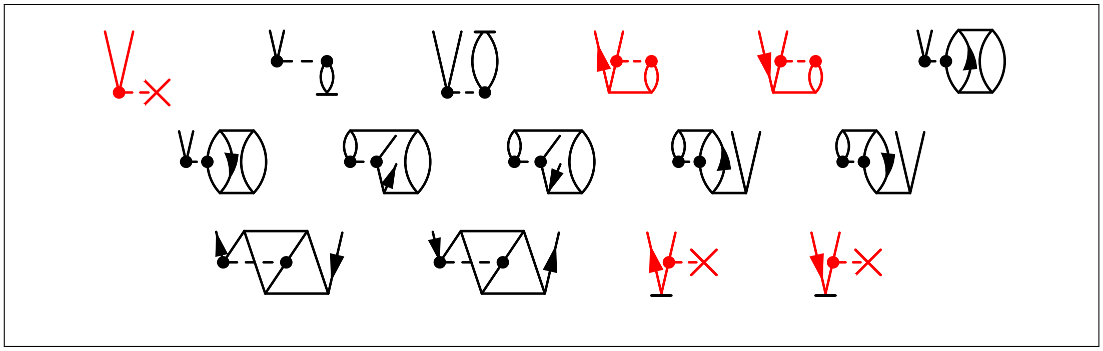

Diagrams S2: Diagrams describing the UCC amplitude equation $\bar{H}_{ai} = 0$. The same conventions for the operators as in diags. 1 are adopted. The red diagrams are those contributing to the UCC2 approximation, while all terms are needed for the UCC3 one.

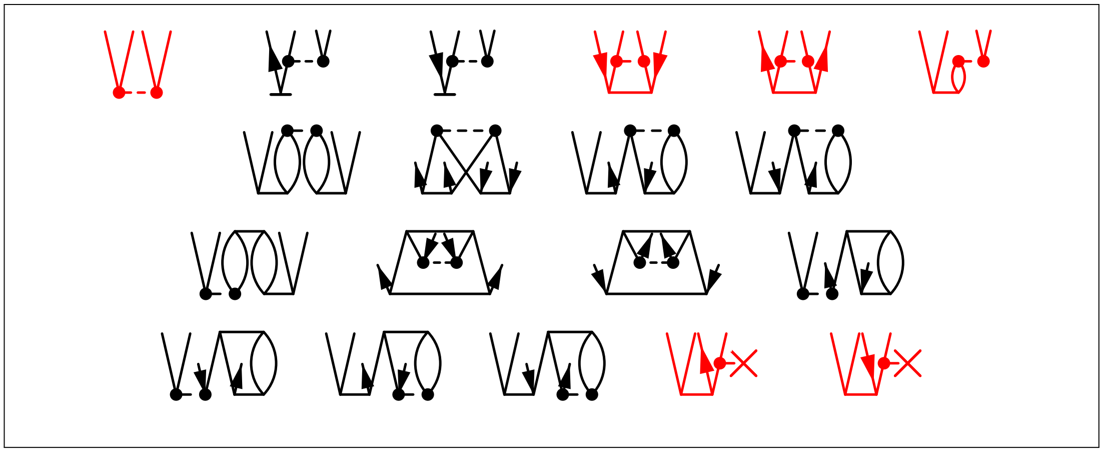

Diagrams S3: Diagrams describing the UCC amplitude equation $\bar{H}_{abij} = 0$. The same conventions for the operators as in diags. 1 are adopted. The red diagrams are those contributing to the UCC2 approximation, while all terms are needed for the UCC3 one.

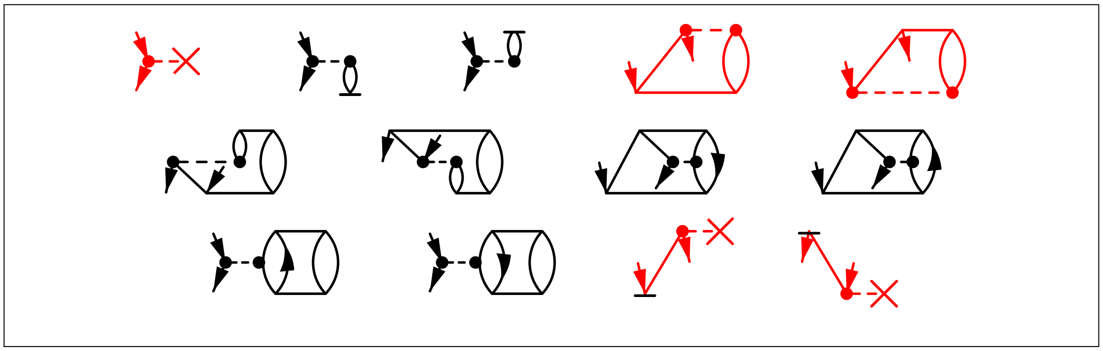

Diagrams S4: Diagrams of the contributions to the transformed Hamiltonian matrix element $\bar{H}_{ij}$. The red diagrams contribute to the UCC2 element $\bar{H}^{(2)}_{ij}$, while all diagrams contribute to the UCC3 block $\bar{H}^{(3)}_{ij}$.

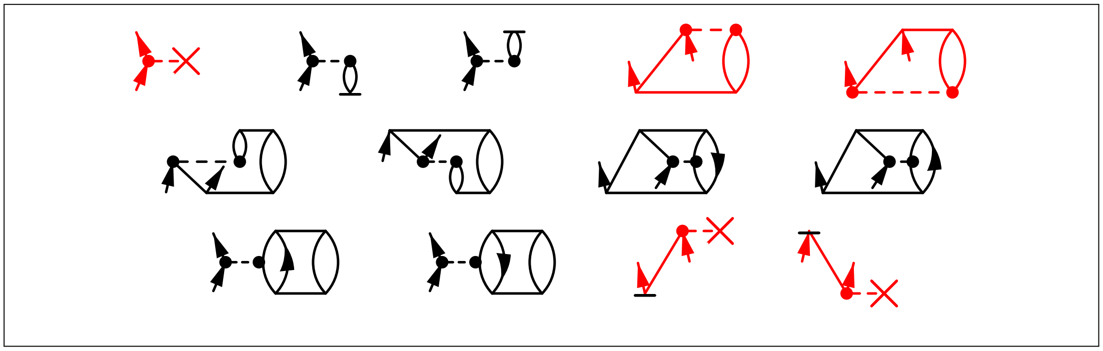

Diagrams S5: Diagrams of the contributions to the transformed Hamiltonian matrix element $\bar{H}_{ab}$. The red diagrams contribute to the UCC2 element $\bar{H}^{(2)}_{ab}$, while all diagrams contribute to the UCC3 block $\bar{H}^{(3)}_{ab}$.

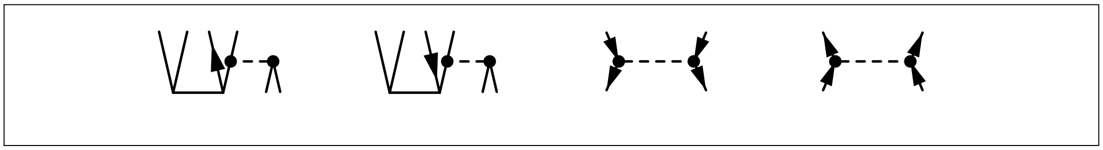

Diagrams S6: Diagrams of the contributions to the transformed Hamiltonian matrix elements for the UCC3 method $\bar{H}^{(1)}_{ibcajk}$, $\bar{H}^{(1)}_{ijkl}$, $\bar{H}^{(1)}_{abcd}$. The corresponding elements for UCC2 are vanishing, as none of these terms is of zeroth order, in order to satisfy $\bar{H}^{(0)}_{ibcajk}$, $\bar{H}^{(0)}_{ijkl}$, $\bar{H}^{(0)}_{abcd}$.

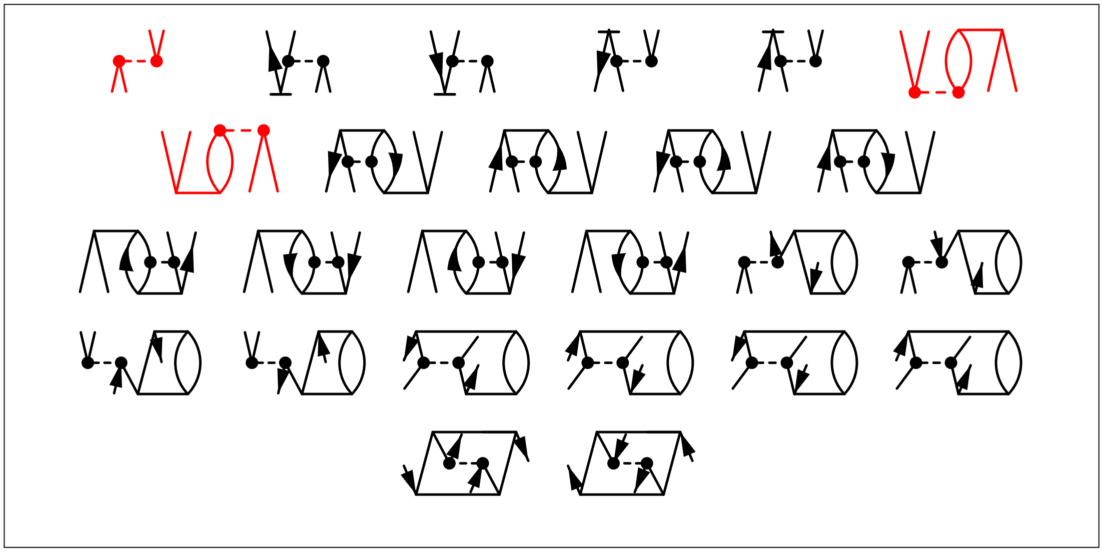

Diagrams S7: Diagrams of the contributions to the transformed Hamiltonian matrix element $\bar{H}_{iabj}$. The red diagrams contribute to the UCC2 element $\bar{H}^{(2)}_{iabj}$, while all diagrams contribute to the UCC3 block $\bar{H}^{(3)}_{iabj}$.

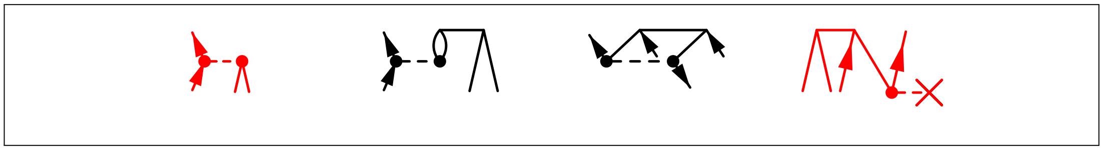

Diagrams S8: Diagrams of the contributions to the transformed Hamiltonian matrix element $\bar{H}_{ciab}$. The red diagrams contribute to the UCC2 element $\bar{H}^{(1)}_{ciab}$, while all diagrams contribute to the UCC3 block $\bar{H}^{(2)}_{ciab}$.

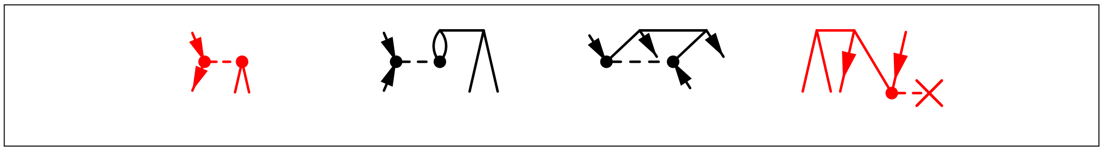

Diagrams S9: Diagrams of the contributions to the transformed Hamiltonian matrix element $\bar{H}_{jkia}$. The red diagrams contribute to the UCC2 element $\bar{H}^{(1)}_{jkia}$, while all diagrams contribute to the UCC3 block $\bar{H}^{(2)}_{jkia}$

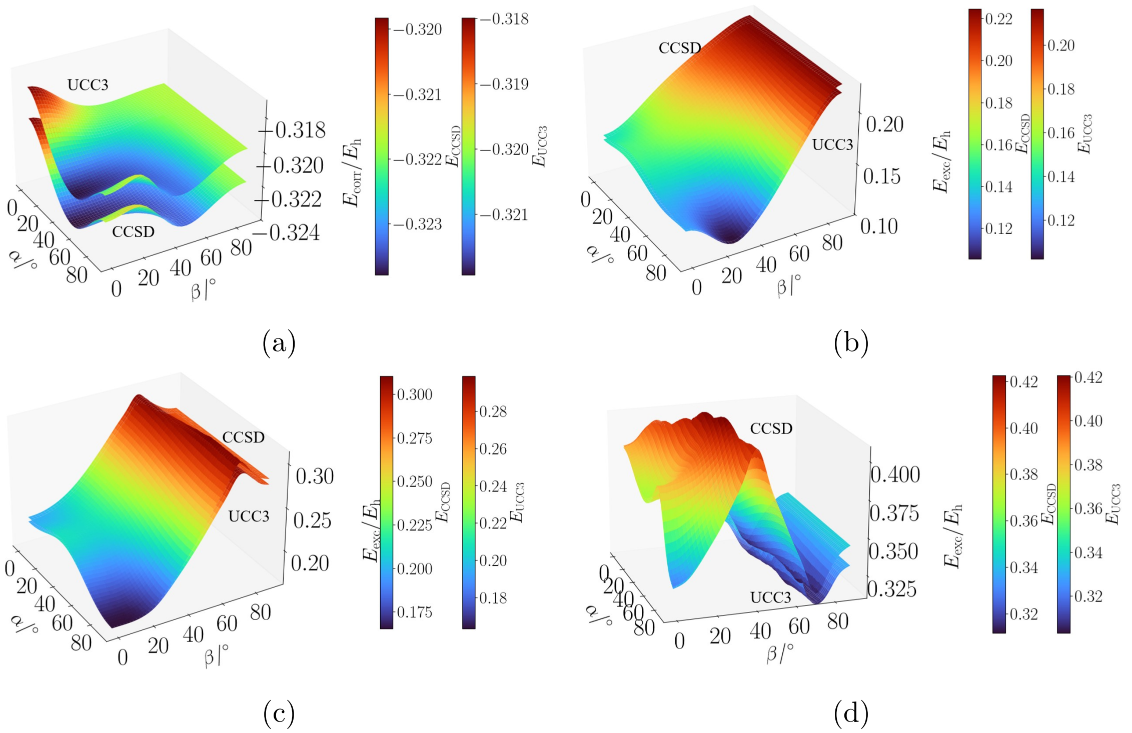

Figure S1: Correlation energy of the ground state (Fig. S1a) and excitation energies of the first three excited states (Figs. S1b, S1c, S1d) of the water molecule in a magnetic field of B=0.5 B_0 as a function of its orientation, as pictured in Fig. 2 of the manuscript, calculated at the ff-CCSD and ff-UCC3 level of theory, using the unc-cc-pVTZ basis set. Note that for CCSD, only the real part of the energy is plotted.

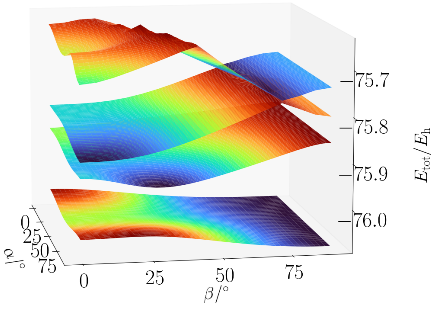

(a) Four lowest-lying singlet states with ff-CCSD.

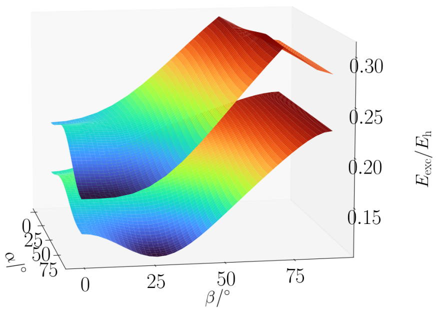

(b) Ψ_1 and Ψ_2 with ff-CCSD.

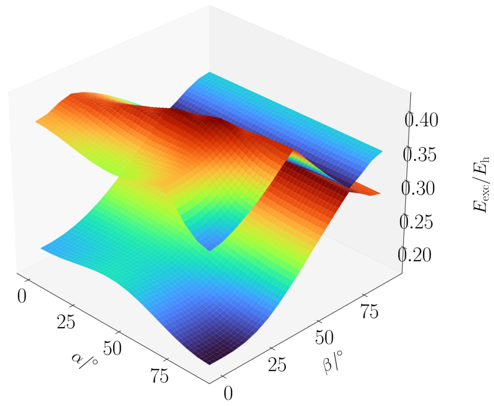

(c) Ψ_2 and Ψ_3 with ff-CCSD.

Figure S2: Real part of the energy surfaces of the ground and first three excited states of the water molecule in a magnetic field of B=0.5 B_0 as a function of its orientation, calculated at the ff-CCSD level of theory (Fig. S2a). In Fig. S2b the excited states Ψ_1 and Ψ_2 are shown, and in Fig. S2c Ψ_2 and Ψ_3 are displayed.

Table S1: Plotted points corresponding to the $1^1\Sigma^+/1^1\Sigma$ state in fig. 1a. The corresponding magnetic field is obtained by $B = 0.05 \times i$.

i	CCSD	UCC3	CCSDT	UCC2
0	-38.0038356753	-38.0022837468	-38.0057417899	-38.0086394109
1	-38.0020202151	-38.0004699229	-38.0039241590	-38.0068149783
2	-37.9965831825	-37.9950377216	-37.9984806837	-38.0013515071
3	-37.9875523495	-37.9860146922	-37.9894393363	-37.9922781333
4	-37.9749731131	-37.9734458929	-37.9768458239	-37.9796423087
5	-37.9589072204	-37.9573926560	-37.9607622825	-37.9635082534
6	-37.9394310931	-37.9379309582	-37.9412655672	-37.9439549924
7	-37.9166338446	-37.9151494794	-37.9184452360	-37.9210741282
8	-37.8906150764	-37.8891474331	-37.8924013196	-37.8949674210
9	-37.8614825792	-37.8600322902	-37.8632420021	-37.8657444345
10	-37.8293499990	-37.8279174563	-37.8310812752	-37.8335201264
11	-37.7943345560	-37.7929199931	-37.7960366520	-37.7984126037
12	-37.7565549085	-37.7551584771	-37.7582270302	-37.7605410316
13	-37.7161291392	-37.7147509755	-37.7177706817	-37.7200237460
14	-37.6731732764	-37.6718135567	-37.6747837793	-37.6769766084
15	-37.6277994594	-37.6264584407	-37.6293785795	-37.6315116186
16	-37.5801150249	-37.5787930735	-37.5816624681	-37.5837358032
17	-37.5302215551	-37.5289191628	-37.5317370872	-37.5337503787
18	-37.4782142284	-37.4769320183	-37.4796976427	-37.4816501797
19	-37.4241815139	-37.4229202360	-37.4256326156	-37.4275233303
20	-37.3682049980	-37.3669655146	-37.3696235915	-37.3714511324

Table S2: Plotted points corresponding to the $1^1\Pi/1^1\Pi_+$ state in fig. 1a. The corresponding magnetic field is obtained by $B = 0.05 \times i$.

i	CCSD	UCC3	CCSDT	UCC2
0	-37.8846233169	-37.8871607984	-37.887682	-37.8711024891
1	-37.8574438530	-37.8599808074	-37.860500	-37.8439119263
2	-37.8259218131	-37.8284572110	-37.828974	-37.8123567087
3	-37.7901056872	-37.7926385693	-37.793149	-37.7764856973
4	-37.7500744313	-37.7526039425	-37.753108	-37.7363785092
5	-37.7059347606	-37.7084601658	-37.708956	-37.6921428614
6	-37.6578175305	-37.6603382082	-37.660824	-37.6439110021
7	-37.6058733842	-37.6083887945	-37.608865	-37.5918353966
8	-37.5502678794	-37.5527775095	-37.553244	-37.5360838433
9	-37.4911763856	-37.4936796743	-37.494137	-37.4768343644
10	-37.4287790011	-37.4312752513	-37.431726	-37.4142700349
11	-37.3632557684	-37.3657440545	-37.366187	-37.3485741106
12	-37.2947824411	-37.2972615201	-37.297702	-37.2799256671
13	-37.2235268956	-37.2259951303	-37.226435	-37.2084959476
14	-37.1496466537	-37.1521019546	-37.152544	-37.1344455136
15	-37.0732866131	-37.0757264008	-37.076176	-37.0579222559
16	-36.9945782019	-36.9969993947	-36.997458	-36.9790602085
17	-36.9136388951	-36.9160379207	-36.916512	-36.8979790776
18	-36.8305723217	-36.8329451512	-36.833438	-36.8147843598
19	-36.7454688831	-36.7478110859	-36.748327	-36.7295679061
20	-36.6584065729	-36.6607133868	-36.661259	-36.6424087971

Table S3: Plotted points corresponding to the $1^1\Pi/11\Pi_-$ state in fig. 1a. The corresponding magnetic field is obtained by $B = 0.05 \times i$.

i	CCSD	UCC3	CCSDT	UCC2
0	-37.8846233169	-37.8871607984	-37.8876813735	-37.8711024891
1	-37.9074438530	-37.9099808074	-37.9105002556	-37.8939119263
2	-37.9259218131	-37.9284572110	-37.9289740000	-37.9123567087
3	-37.9401056872	-37.9426385693	-37.9431493519	-37.9264856973
4	-37.9500744313	-37.9526039425	-37.9531076400	-37.9363785092
5	-37.9559347606	-37.9584601658	-37.9589554083	-37.9421428614
6	-37.9578175306	-37.9603382082	-37.9608240737	-37.9439110021
7	-37.9558733842	-37.9583887945	-37.9588648668	-37.9418353966
8	-37.9502678794	-37.9527775095	-37.9532439180	-37.9360838433
9	-37.9411763856	-37.9436796743	-37.9441371158	-37.9268343644
10	-37.9287790011	-37.9312752513	-37.9317249941	-37.9142700349
11	-37.9132557684	-37.9157440545	-37.916187000	-37.8985741106
12	-37.8947824411	-37.8972615201	-37.8977018812	-37.8799256671
13	-37.8735268956	-37.8759951303	-37.8764348356	-37.8584959476
14	-37.8496466537	-37.8521019546	-37.8525443046	-37.8344455136
15	-37.8232866131	-37.8257264008	-37.8261750936	-37.8079222559
16	-37.7945782019	-37.7969993947	-37.7974584570	-37.7790602085
17	-37.7636388951	-37.7660379207	-37.7665116632	-37.7479790776
18	-37.7305723217	-37.7329451512	-37.7334381065	-37.7147843598
19	-37.6954688831	-37.6978110859	-37.6983279513	-37.6795679061
20	-37.6584065729	-37.6607133868	-37.6612589656	-37.6424087971

Table S4: Plotted points corresponding to the $1^1\Delta/1^1\Delta_{-2}$ state in fig. 1a. The corresponding magnetic field is obtained by $B = 0.05 \times i$.

i	CCSD	UCC3	CCSDT	UCC2
0	-37.7151563326	-37.8051510322	-37.7501246972	
1	-37.7625611120	-37.8525505314	-37.7975382891	
2	-37.8047979528	-37.8947714683	-37.8398014280	
3	-37.8419336833	-37.9318804539	-37.8769805049	
4	-37.8740774319	-37.9639861703	-37.9091838611	
5	-37.9013772879	-37.9912359258	-37.9365583717	
6	-37.9240157228	-38.0138109612	-37.9592847783	
7	-37.9422038868	-38.0319206490	-37.9775719053	
8	-37.9561749505	-38.0457957751	-37.9916499477	
9	-37.9661767710	-38.0556811972	-38.0017631305	
10	-37.9724641838	-38.0618281857	-38.0081620527	
11	-37.9752913207	-38.0644868411	-38.0110961240	
12	-37.9749044044	-38.0638990259	-38.0108065364	
13	-37.9715353559	-38.0602921317	-38.0075200912	
14	-37.9653969299	-38.0538743719	-38.0014445731	
15	-37.9566786962	-38.0448309138	-37.9927649525	
16	-37.9455452003	-38.0333221602	-37.9816417349	
17	-37.9321352823	-38.0194831640	-37.9682104100	
18	-37.9165627050	-38.0034243256	-37.9525821467	
19	-37.8989178710	-37.9852331568	-37.9348455185	
20	-37.8792701326	-37.9649766182	-37.9150687688	

Table S5: Plotted points corresponding to the $1^1\Sigma^+/1^1A$ state in fig. 1b. The corresponding magnetic field is obtained by $B = 0.05 \times i$.

i	CCSD	UCC3	CCSDT	UCC2
0	-38.0038356753	-38.0022837486	-38.005742	-38.0086394109
1	-38.0045018476	-38.0038013492	-38.006431	-38.0086599805
2	-38.0067938883	-38.0066442067	-38.008773	-38.0101095359
3	-38.0112521485	-38.0108956679	-38.013251	-38.0142038636
4	-38.0178571136	-38.0172247069	-38.019814	-38.0206579113
5	-38.0257389289	-38.0249694083	-38.027621	-38.0283942941
6	-38.0336686385	-38.0328522575	-38.035485	-38.0361721918
7	-38.0405351795	-38.0397065852	-38.042318	-38.0429080660
8	-38.0455196590	-38.0446887763	-38.047300	-38.0477901009
9	-38.0481009504	-38.0472696934	-38.049901	-38.0502910636
10	-38.0479970448	-38.0471654319	-38.049832	-38.0501212567
11	-38.0450949592	-38.0442628644	-38.046972	-38.0471617520
12	-38.0393905687	-38.0385579843	-38.041316	-38.0414045747
13	-38.0309442832	-38.0301114176	-38.032919	-38.0329078289
14	-38.0198511362	-38.0190182311	-38.021878	-38.0217647996
15	-38.0062199399	-38.0053873368	-38.008298	-38.0080834419
16	-37.9901608132	-37.9893288940	-37.992290	-37.9919732808
17	-37.9717773055	-37.9709464789	-37.973956	-37.9735374518
18	-37.9511621122	-37.9503328114	-37.953390	-37.9528683396
19	-37.9283950542	-37.9275677181	-37.930671	-37.9300457085
20	-37.9035432569	-37.902718346	-37.90586	-37.9051365161

Table S6: Plotted points corresponding to the $1^1\Pi/2^1A$ state in fig. 1b. The corresponding magnetic field is obtained by $B = 0.05 \times i$.

i	CCSD	UCC3	CCSDT	UCC2
0	-37.8846233187	-37.8871608000	-37.887682	-37.8711024891
1	-37.9039161615	-37.9107559276	-37.906770	-37.8880628562
2	-37.9181158361	-37.9265242594	-37.920624	-37.8988140547
3	-37.9273653986	-37.9325485728	-37.929552	-37.9067264734
4	-37.9329939957	-37.9355591854	-37.934845	-37.9129183606
5	-37.9363174442	-37.9380534303	-37.937971	-37.9172299018
6	-37.9373739052	-37.9390533253	-37.939052	-37.9189718909
7	-37.9354955705	-37.9372524678	-37.937293	-37.9174991450
8	-37.9302166528	-37.9320622810	-37.932131	-37.9124739547
9	-37.9214399595	-37.9233848941	-37.923448	-37.9038523395
10	-37.9092872701	-37.9113456609	-37.911368	-37.8917697744
11	-37.8939732943	-37.8961606165	-37.896113	-37.8764445983
12	-37.8757449086	-37.8780800355	-37.877937	-37.8581242895
13	-37.8548583872	-37.8573670413	-37.857099	-37.8370618156
14	-37.8315739259	-37.8342920914	-37.833868	-37.8135075590
15	-37.8061560301	-37.8091329575	-37.808511	-37.7877082871
16	-37.7788775298	-37.7821777202	-37.781309	-37.7599089940
17	-37.7500228703	-37.7537256481	-37.752553	-37.7303555787
18	-37.7198877592	-37.7240818711	-37.722550	-37.6992966006
19	-37.6887701281	-37.6935403087	-37.691606	-37.6669820623
20	-37.6569485470	-37.6623530481	-37.660004	-37.6336566942

Table S7: Plotted points corresponding to the $1^1\Pi/3^1A$ state in fig. 1b. The corresponding magnetic field is obtained by $B = 0.05 \times i$.

i	CCSD	UCC3	CCSDT	UCC2
0	-37.8846233187	-37.8871608000	-37.887682	-37.8711024891
1	-37.8608133391	-37.8679647809	-37.863614	-37.8444910812
2	-37.8328643179	-37.8741224319	-37.835154	-37.8107376858
3	-37.8091320000	-37.8919530235	-37.845509	-37.7736617939
4	-37.8201548799	-37.8985644290	-37.858160	-37.7353215862
5	-37.8201613534	-37.8948746608	-37.859593	-37.6964945128
6	-37.8104888730	-37.8827368547	-37.851124	-37.6571472925
7	-37.7934603704	-37.8641627087	-37.835066	-37.6173920863
8	-37.7709619911	-37.8405646775	-37.813314	-37.5955669144
9	-37.7442991568	-37.8128421152	-37.787112	-37.6014012700
10	-37.7145976638	-37.7816138368	-37.757330	-37.6051975230
11	-37.6839104852	-37.7474235846	-37.724843	-37.6056731036
12	-37.6592127420	-37.7111020943	-37.691435	-37.6028417991
13	-37.6458943509	-37.6757201844	-37.663121	-37.5968244715
14	-37.6351365006	-37.6522697126	-37.645725	-37.5877491226
15	-37.6228110679	-37.6372802786	-37.631559	-37.5757244320
16	-37.6080113335	-37.6215798887	-37.616080	-37.5608277141
17	-37.5904453832	-37.6033718570	-37.598179	-37.5430986197
18	-37.5699442433	-37.5822003116	-37.577465	-37.5225369601
19	-37.5463597117	-37.5578422083	-37.553708	-37.4991055463
20	-37.5195556986	-37.5301584081	-37.526737	-37.4727396556

Table S8: Plotted points corresponding to the $1^1\Delta/4^1A$ state in fig. 1b. The corresponding magnetic field is obtained by $B = 0.05 \times i$.

i	CCSD	UCC3	CCSDT	UCC2
0	-37.7151563347	-37.8051510340	-37.750125	
1	-37.7547435421	-37.8446625993	-37.789986	
2	-37.7863631152	-37.8436559483	-37.822195	
3	-37.8003090626	-37.8106840839	-37.802704	
4	-37.7645135619	-37.7731107163	-37.766868	
5	-37.7248950112	-37.7329093458	-37.727896	
6	-37.6824868177	-37.6901932810	-37.686209	
7	-37.6381577970	-37.6458560740	-37.642563	
8	-37.6297480976	-37.6466231702	-37.638204	
9	-37.6373438945	-37.6542551874	-37.645814	
10	-37.6407400022	-37.6584346977	-37.649703	
11	-37.6383888127	-37.6589727401	-37.649508	
12	-37.6237750589	-37.6554077145	-37.643911	
13	-37.5919123843	-37.6450077730	-37.627289	
14	-37.5519787993	-37.6170877766	-37.594183	
15	-37.5084251508	-37.5753940196	-37.552583	
16	-37.4624612435	-37.5293553393	-37.507329	
17	-37.4147230359	-37.4810420210	-37.459851	
18	-37.3665160594	-37.4313202082	-37.411103	
19	-37.3353882121	-37.3815142842	-37.363662	
20	-37.3131717822	-37.3372248148	-37.328846	

Table S9: Plotted points corresponding to the $1^1\Sigma^+/1^1A$ state in fig. 1c. The corresponding magnetic field is obtained by $B = 0.05 \times i$.

i	CCSD	UCC3	CCSDT	UCC2
0	-38.0038356753	-38.0022837468	-38.005742	-38.0086394498
1	-38.0090876946	-38.0084379458	-38.011051	-38.0127453444
2	-38.0222167876	-38.0213423942	-38.024221	-38.0253563020
3	-38.0386027837	-38.0376066831	-38.040571	-38.0416188217
4	-38.0549147043	-38.0538948265	-38.056826	-38.0578563555
5	-38.0692900974	-38.0682745367	-38.071150	-38.0721657473
6	-38.0807453786	-38.0797401891	-38.082565	-38.0835621008
7	-38.0887822883	-38.0877875205	-38.090573	-38.0915471569
8	-38.0931809932	-38.0921951808	-38.094952	-38.0959001720
9	-38.0938880574	-38.0929095172	-38.095615	-38.0965667548
10	-38.0909514199	-38.0899785176	-38.092699	-38.0935940409
11	-38.0844796457	-38.0835109221	-38.086222	-38.0870902007
12	-38.0746153779	-38.0736495216	-38.076358	-38.0771976246
13	-38.0615173191	-38.0605531457	-38.063263	-38.0640748693
14	-38.0453481510	-38.0443845778	-38.047101	-38.0478846441
15	-38.0262672433	-38.0253032591	-38.028031	-38.0287863284
16	-38.0044264630	-38.0034610934	-38.006204	-38.0069319373
17	-37.9799687762	-37.9790010452	-37.981764	-37.9824645054
18	-37.9530281245	-37.9520570107	-37.954843	-37.9555181682
19	-37.9237304102	-37.9227547932	-37.925570	-37.9262189849
20	-37.8921945972	-37.8912132388	-37.894062	-37.8946861206

Table S10: Plotted points corresponding to the $1^1\Pi/2^1A$ state in fig. 1c. The corresponding magnetic field is obtained by $B = 0.05 \times i$.

i	CCSD	UCC3	CCSDT	UCC2
0	-37.8846233169	-37.8871607982	-37.887682	-37.8711024965
1	-37.8949030678	-37.9018407787	-37.897550	-37.8774880559
2	-37.9018449357	-37.9059606423	-37.904094	-37.8827532773
3	-37.9067816940	-37.9092772836	-37.908748	-37.8880234305
4	-37.9096096743	-37.9114778092	-37.911390	-37.8914707527
5	-37.9097113093	-37.9113501593	-37.911388	-37.8920556519
6	-37.9066230473	-37.9081882843	-37.908250	-37.8892934820
7	-37.9001359882	-37.9016920232	-37.901746	-37.8830152952
8	-37.8902318465	-37.8918105439	-37.891850	-37.8732320311
9	-37.8770144537	-37.8786381263	-37.878631	-37.8600604343
10	-37.8606655165	-37.8623569830	-37.862360	-37.8436826246
11	-37.8414197869	-37.8432082268	-37.843188	-37.8243239029
12	-37.8195529442	-37.8214787449	-37.821429	-37.8022407497
13	-37.7953767152	-37.7974963809	-37.797400	-37.7777147830
14	-37.7692378096	-37.7716287295	-37.771460	-37.7510503883
15	-37.7415177413	-37.7442812663	-37.743995	-37.7225744455
16	-37.7126282905	-37.7158878473	-37.715421	-37.6926363435
17	-37.6829961651	-37.6868859343	-37.686157	-37.6616052048
18	-37.6530277974	-37.6576695379	-37.656584	-37.6298589140
19	-37.6230515619	-37.6285253223	-37.626998	-37.5977571668
20	-37.5932501313	-37.599574888	-37.597541	-37.5655923930

Table S11: Plotted points corresponding to the $1^1\Delta/4^1A$ state in fig. 1c. The corresponding magnetic field is obtained by $B = 0.05 \times i$.

i	CCSD	UCC3	CCSDT	UCC2
0	-37.8846233169	-37.8871607982	-37.887682	-37.8711024965
1	-37.8695596836	-37.8776248549	-37.871981	-37.8494719963
2	-37.8491172800	-37.8561681000	-37.850792	-37.8224092316
3	-37.8246039140	-37.8325654437	-37.826302	-37.7952524410
4	-37.7978496631	-37.8154538550	-37.801317	-37.7670219107
5	-37.7700356854	-37.7985298927	-37.778284	-37.7367442442
6	-37.7418239734	-37.7785126423	-37.756241	-37.7040744678
7	-37.7138535727	-37.7555151245	-37.733274	-37.6695506668
8	-37.6874144140	-37.7301928639	-37.709312	-37.6356868238
9	-37.6651202899	-37.7037118286	-37.685855	-37.6100712507
10	-37.6491492178	-37.6782946999	-37.665644	-37.5981958250
11	-37.6373742657	-37.6569723371	-37.649727	-37.5910413560
12	-37.6261850162	-37.6400446426	-37.635934	-37.5829719808
13	-37.6136674696	-37.6245600510	-37.621782	-37.5727164080
14	-37.5991335645	-37.6082278159	-37.606042	-37.5601325996
15	-37.5823939031	-37.5900612366	-37.588268	-37.5453556080
16	-37.5634230068	-37.5696911505	-37.568334	-37.5285394898
17	-37.5422198779	-37.5470034324	-37.546243	-37.5097434730
18	-37.5187614929	-37.5220318648	-37.522038	-37.4888884036
19	-37.4930198751	-37.4949620343	-37.495805	-37.4657767524
20	-37.4650204477	-37.4661581292	-37.467682	-37.4401677163

Table S12: Plotted points corresponding to the $1^1\Pi/1^1\Pi_+$ state in fig. 1c. The corresponding magnetic field is obtained by $B = 0.05 \times i$.

i	CCSD	UCC3	CCSDT	UCC2
0	-37.7151563329	-37.8051510322	-37.750125	
1	-37.7344707001	-37.8226286122	-37.770132	
2	-37.7425469435	-37.8250359079	-37.779421	
3	-37.7400254660	-37.8154538550	-37.777966	
4	-37.7283754587	-37.7904752575	-37.765829	
5	-37.7091929613	-37.7575647355	-37.742966	
6	-37.6838704576	-37.7215537069	-37.712158	
7	-37.6538464151	-37.6838513950	-37.677100	
8	-37.624980,	-37.6473176177	-37.640987	
9	-37.5972988790	-37.6031077769	-37.613686	
10	-37.5789222111	-37.5818659871	-37.598240	
11	-37.5628697046	-37.5680592702	-37.575404	
12	-37.5508375666	-37.5558572523	-37.558485	
13	-37.5380840921	-37.5461275974	-37.545679	
14	-37.5234189071	-37.5319743442	-37.531374	
15	-37.5065462366	-37.5165100517	-37.515078	
16	-37.4873154488	-37.4992560105	-37.496585	
17	-37.4655838477	-37.4800114290	-37.475702	
18	-37.4412300274	-37.4585625386	-37.452239	
19	-37.4141995272	-37.4346779686	-37.426067	
20	-37.3845365243	-37.4081349226	-37.397162	

Table S13: Plotted points corresponding to the $1^1\Sigma^+/1^1A'$ state in fig. 1d. The corresponding magnetic field is obtained by $B = 0.05 \times i$.

i	CCSD	UCC3	CCSDT	UCC2
0	-38.0038356753	-38.0022837468	-38.005742	-38.0086394109
1	-38.0112243885	-38.0105148921	-38.013200	-38.0147641157
2	-38.0285131804	-38.0275470735	-38.030514	-38.0316423963
3	-38.0486202124	-38.0475786139	-38.050576	-38.0516607098
4	-38.0676108749	-38.0665633732	-38.069512	-38.0705987780
5	-38.0836962895	-38.0826570109	-38.085552	-38.0866412842
6	-38.0960746065	-38.0950448623	-38.097895	-38.0989834503
7	-38.1044110799	-38.1033894747	-38.106204	-38.1072892315
8	-38.1086133033	-38.1075979823	-38.110384	-38.1114654456
9	-38.1087238169	-38.1077129503	-38.110477	-38.1115543133
10	-38.1048623194	-38.1038541556	-38.106600	-38.1076755373
11	-38.0971910205	-38.0961839980	-38.098917	-38.0999916229
12	-38.0858927638	-38.0848853364	-38.087611	-38.0886856554
13	-38.0711564342	-38.0701471658	-38.072870	-38.0739469078
14	-38.0531683140	-38.0521558268	-38.054879	-38.0559620935
15	-38.0321075410	-38.0310905247	-38.033818	-38.0349108578
16	-38.0081446668	-38.0071218156	-38.009857	-38.0109643514
17	-37.9814420997	-37.9804120895	-37.983159	-37.9842856931
18	-37.9521555321	-37.9511169153	-37.953879	-37.9550314380
19	-37.9204357562	-37.9193869363	-37.922169	-37.9233533566
20	-37.8864302314	-37.8853693298	-37.888174	-37.8894000438

Table S14: Plotted points corresponding to the $1^1\Pi/1^1A''$ state in fig. 1d. The corresponding magnetic field is obtained by $B = 0.05 \times i$.

i	CCSD	UCC3	CCSDT	UCC2
0	-37.8846233169	-37.8871607982	-37.887682	-37.8711024891
1	-37.8861002182	-37.8902746641	-37.888980	-37.8705673920
2	-37.8894390248	-37.8923109636	-37.891953	-37.8730516482
3	-37.8924595066	-37.8945877828	-37.894653	-37.8760862763
4	-37.8934939860	-37.8952598175	-37.895464	-37.8773105660
5	-37.8916047070	-37.8931778116	-37.893425	-37.8756235982
6	-37.8863343689	-37.8877949221	-37.888051	-37.8705389307
7	-37.8775056275	-37.8788947611	-37.879146	-37.8618782045
8	-37.8651050913	-37.8664460100	-37.866689	-37.8496315232
9	-37.8492190078	-37.8505265537	-37.850763	-37.8338881299
10	-37.8299961449	-37.8312816176	-37.831514	-37.8147983767
11	-37.8076249674	-37.8088989922	-37.809135	-37.7925505854
12	-37.7823187768	-37.7835933331	-37.783845	-37.7673557471
13	-37.7543054674	-37.7555958071	-37.755878	-37.7394369496
14	-37.7238205184	-37.7251472086	-37.725477	-37.7090220129
15	-37.6911019915	-37.6924934356	-37.692892	-37.6763383024
16	-37.6563867897	-37.6578826150	-37.658372	-37.6416088076
17	-37.6199076656	-37.6215637441	-37.622168	-37.6050488073
18	-37.5818912801	-37.5837877105	-37.584520	-37.5668628117
19	-37.5425585057	-37.5448129553	-37.545670	-37.5272419419
20	-37.5021285396	-37.5049188412	-37.505857	-37.5203661746

Table S15: Plotted points corresponding to the $1^1\Pi/2^1A'$ state in fig. 1d. The corresponding magnetic field is obtained by $B = 0.05 \times i$.

i	CCSD	UCC3	CCSDT	UCC2
0	-37.8846233169	-37.8871607982	-37.887682	-37.8711024891
1	-37.8782692913	-37.8884838588	-37.880252	-37.8547059879
2	-37.8619096784	-37.8686589300	-37.863078	-37.8319465408
3	-37.8405687177	-37.8455108491	-37.841618	-37.8093057200
4	-37.8163047964	-37.8210039705	-37.817594	-37.7849308235
5	-37.7894501423	-37.7947787803	-37.791157	-37.7578249887
6	-37.7602801960	-37.7667507053	-37.762500	-37.7279763440
7	-37.7298141225	-37.7376209780	-37.732580	-37.6964613899
8	-37.7005494976	-37.7092301180	-37.703761	-37.6662060211
9	-37.6769000775	-37.6849714049	-37.680292	-37.6425530402
10	-37.6619552868	-37.6681746108	-37.665372	-37.6285342237
11	-37.6530851074	-37.6577299557	-37.656550	-37.6202430051
12	-37.6461855436	-37.6498809099	-37.649709	-37.6133091045
13	-37.6391919726	-37.6423269842	-37.642762	-37.6059226451
14	-37.6314050541	-37.6342024194	-37.635009	-37.5975877834
15	-37.6225995724	-37.6252022039	-37.626229	-37.5881865520
16	-37.6126439937	-37.6151591517	-37.616293	-37.5776305157
17	-37.6013879256	-37.6039100600	-37.605058	-37.5657742077
18	-37.5886518210	-37.5912753531	-37.592350	-37.5524219072
19	-37.5742456419	-37.5770722892	-37.577988	-37.5373567539
20	-37.5579881664	-37.5611307854	-37.561796	-37.4863623242

Table S16: Plotted points corresponding to the $1^1\Delta/3^1A'$ state in fig. 1d. The corresponding magnetic field is obtained by $B = 0.05 \times i$.

i	CCSD	UCC3	CCSDT	UCC2
0	-37.7151563329	-37.8051510322	-37.750125	
1	-37.7087625689	-37.7959564961	-37.744673	
2	-37.6916348748	-37.7725517117	-37.729849	
3	-37.6699589709	-37.7467538382	-37.710922	
4	-37.6490850179	-37.7229161829	-37.691239	
5	-37.6279605875	-37.6993952209	-37.670166	
6	-37.6049519624	-37.6743087311	-37.646764	
7	-37.5824979485	-37.6476552852	-37.621581	
8	-37.5800011860	-37.6226168533	-37.600374	
9	-37.5709294618	-37.6030775070	-37.585637	
10	-37.5478228414	-37.5813606651	-37.562949	
11	-37.5142776881	-37.5524494839	-37.531052	
12	-37.4765307489	-37.5187592807	-37.494404	
13	-37.4415637865	-37.4830350029	-37.456545	
14	-37.4170677924	-37.4471543161	-37.422952	
15	-37.3961410320	-37.4128594655	-37.399304	
16	-37.3742417092	-37.3831275758	-37.377190	
17	-37.3507706781	-37.3577312314	-37.353831	
18	-37.3256669873	-37.3323991139	-37.328924	
19	-37.2989056714	-37.3058419613	-37.302401	
20	-37.2704481746	-37.2777987158	-37.274212	

Table S17: Real part of the energies of the ground and excited states of $B(OH)_3$, at the CC3 level of theory, plotted in figs. 8-9.

B	$^1A'$	$^1E_1'$	$^1E_2'$	$^1A''$	$^1E_1''$	$^1E_2''$	
0.00	-252.1615732	0.3541826	0.3622465	0.3622465	0.3311491	0.2989578	0.2989578
0.05	-252.1512566	0.3580695	0.3667007	0.3564556	0.3262937	0.3045107	0.3031327
0.10	-252.1202673	0.3607525	0.3560856	0.3532824	0.3253830	0.3114830	0.3148048
0.15	-252.0686536	0.3518518	0.3377184	0.3463282	0.3275221	0.3138453	0.3271029
0.20	-251.9969309	0.3317732	0.3148731	0.3342740	0.3247675	0.3145165	0.3367404
0.25	-251.9059170	0.2959661	0.2930608	0.3186232	0.3142848	0.3183387	0.3152947
0.30	-251.7965939	0.2524310	0.2711955	0.2958935	0.2982821	0.3247321	0.2826686
0.35	-251.6702794	0.2094421	0.2438151	0.2633166	0.2822553	0.3267626	0.2503777
0.40	-251.5292586	0.1718219	0.2131399	0.2278143	0.2706460	0.3195279	0.2184390
0.45	-251.3781332	0.1422746	0.1870509	0.1957645	0.2548209	0.2858269	0.1868204
0.50	-251.2237622	0.1225633	0.1711574	0.1709015	0.2321578	0.2581311	0.1559749
0.55	-251.0746116	0.1124815	0.1675448	0.1557056	0.2160635	0.2286919	0.1266295
0.60	-250.9380432	0.1090590	0.1721782	0.1441894	0.2080009	0.2012226	0.0995288
0.65	-250.8173692	0.1085530	0.1553920	0.1333382	0.2078458	0.1811276	0.0750664
0.70	-250.7127914	0.1112902	0.1457942	0.1306666		0.1703819	0.0537118
0.75	-250.6236414	0.1210020	0.1420161	0.1379773		0.1674236	0.0370646
0.80	-250.5483272	0.1408681	0.1456565	0.1553567	0.2123631	0.1706537	0.0279712

Table S18: Real part of the energies of the ground and excited states of $B(OH)_3$, at the CCSD level of theory, plotted in figs. 8-9.

B	$^1A'$	$^1E_1'$	$^1E_2'$	$^1A''$	$^1E_1''$	$^1E_2''$	
0.00	-252.1338335	0.3563775	0.3643064	0.3643064	0.3325524	0.3018311	0.3018311
0.05	-252.1235252	0.3600837	0.3680537	0.3585482	0.3278731	0.3068540	0.3062639
0.10	-252.0925679	0.3620730	0.3581307	0.3552980	0.3272641	0.3138190	0.3178197
0.15	-252.0410165	0.3533838	0.3408729	0.3482647	0.3299106	0.3168528	0.3296974
0.20	-251.969379	0.3342771	0.3184873	0.3362435	0.3274795	0.3180631	0.3390395
0.25	-251.8784594	0.2997759	0.2965897	0.3206986	0.3175134	0.3220579	0.3193303
0.30	-251.7692329	0.2572341	0.2744005	0.2983150	0.3024007	0.3281348	0.2867486
0.35	-251.6430023	0.2149598	0.2473360	0.2665971	0.2871499	0.3296112	0.2545856
0.40	-251.5019856	0.1779373	0.2175239	0.2321141	0.2761418	0.3177958	0.2228791
0.45	-251.3505749	0.1489827	0.1921470	0.2012046	0.2603470	0.2928293	0.1916063
0.50	-251.1953308	0.1298568	0.1767658	0.1772342	0.2380652	0.2671537	0.1611644
0.55	-251.044602	0.1201260	0.1735669	0.1624989	0.2227904	0.2409856	0.1319525
0.60	-250.9062907	0.1163489	0.1782811	0.1534698	0.2160388	0.2160962	0.1042333
0.65	-250.784718	0.1147373	0.1732017	0.1452620	0.2171219	0.1959226	0.0783819
0.70	-250.6806567	0.1165736	0.1600137	0.1419997	0.2237878	0.1830113	0.0555349
0.75	-250.5931261	0.1263525	0.1514668	0.1479438	0.2331452	0.1783444	0.0380361
0.80	-250.5197375	0.1468881	0.1527658	0.1639495	0.2432309	0.1821163	0.0288210

Table S19: Energies of the ground and excited states of $B(OH)_3$, at the UCC3 level of theory, plotted in figs. 8-9.

B	$^1A'$	$^1E'_1$	$^1E'_2$	$^1A''$	$^1E''_1$	$^1E''_2$	
0.00	-252.12968	0.3665601	0.3737871	0.3737871	0.3388748	0.3081290	0.3081290
0.05	-252.11938	0.3696815	0.3771565	0.3677284	0.3341629	0.3127302	0.3124876
0.10	-252.08842	0.3703942	0.3662751	0.3635343	0.3327917	0.3190020	0.3236196
0.15	-252.03687	0.3604577	0.3479972	0.3554441	0.3342886	0.3213822	0.3347876
0.20	-251.96524	0.3402480	0.3247925	0.3422714	0.3310593	0.3219120	0.3428780
0.25	-251.87432	0.3052962	0.3017865	0.3253862	0.3206898	0.3249537	0.3220260
0.30	-251.76509	0.2620728	0.2783526	0.3018212	0.3052415	0.3298871	0.2893802
0.35	-251.63884	0.2188194	0.2503444	0.2692200	0.2896441	0.3303710	0.2568473
0.40	-251.49777	0.1804150	0.2195323	0.2333741	0.2781906	0.3229538	0.2243689
0.45	-251.34628	0.1493312	0.1925038	0.2002749	0.2621804	0.2939249	0.1916214
0.50	-251.19091	0.1267819	0.1743396	0.1733468	0.2384567	0.2641126	0.1586962
0.55	-251.03981	0.1135000	0.1693765	0.1558691	0.2209783		0.1276036
0.60	-250.90276		0.1670595	0.1230165	0.2076759	0.2177013	0.1031910
0.65	-250.79022	0.0832747	0.1582047	0.0956200	0.2073437		0.0765885
0.70	-250.69213	0.0876877	0.1444522	0.1018524	0.2093140		0.0492123
0.75	-250.59834	0.1176901	0.1429978	0.1350481	0.2230153	0.1708424	0.0326696
0.80	-250.51846	0.1435741	0.1443495	0.1607560	0.2379496	0.1677649	0.0230444

Table S20: Energies of the ground and excited states of $B(OH)_3$, at the CISD level of theory, plotted in figs. 8-9.

B	$^1A'$	$^1E'_1$	$^1E'_2$	$^1A''$	$^1E''_1$	$^1E''_2$	
0.00	-252.1338335	0.3563775	0.3643064	0.36430643	0.3325524	0.3018311	0.3018311
0.05	-252.1235974	0.3601461	0.3685908	0.35780056	0.3283769	0.3070667	0.3052452
0.10	-252.0928975	0.3629264	0.3573669	0.35368339	0.3272861	0.3130295	0.3158459
0.15	-252.0418074	0.3539828	0.3385164	0.34645595	0.3289071	0.3148656	0.3276228
0.20	-251.9707959	0.3332930	0.3153941	0.33487801	0.3256802	0.3153212	0.3286663
0.25	-251.8806127	0.2973552	0.2930715	0.31991674	0.3148537	0.3186736	0.2978657
0.30	-251.7721939	0.2537368	0.2713456	0.29815431	0.2989116	0.3246462	0.2659881
0.35	-251.6467860	0.2105117	0.2456587	0.26633430	0.2830924	0.3271482	0.2342779
0.40	-251.5063558	0.1722818	0.2161478	0.23035773	0.2703330	0.3215726	0.2027304
0.45	-251.3546161	0.1413072	0.1896430	0.19607470	0.2447551	0.2799579	0.1711801
0.50	-251.1975313	0.1188774	0.1721207	0.16650938	0.2220394	0.2536825	0.1396970
0.55	-251.0440596	0.1045684	0.1665905	0.14379399	0.2067602	0.2251411	0.1086138
0.60	-250.9053110	0.0954131	0.1696122	0.12344879	0.2011657	0.1964149	0.0791192
0.65	-250.7896868	0.0887694	0.1495996	0.10688513	0.2050039	0.1740078	0.0531745
0.70	-250.6984172	0.0890721	0.1358228	0.10462406	0.2146956	0.1626156	0.0322392
0.75	-250.6270310	0.1013251	0.1281018	0.11605884	0.2246224	0.1618413	0.0177084
0.80	-250.5692130	0.1257716	0.1307909	0.13771910	0.2356567	0.1698333	0.0116680

B	$^1A'$	$^1E'_1$	$^1E'_2$	$^1A''$	$^1E''_1$	$^1E''_2$	
0.00	0.000000000	0.000000000	3.45180E-06	-3.45330E-06	-5E-10	-1.190740E-05	1.190740E-05
0.05	0.000001095	-1.03408E-05	3.55369E-06	1.28712E-05	6.78483E-06	9.858962E-06	-1.095680E-05
0.10	0.000002108	-1.09544E-05	6.08940E-06	1.43772E-05	2.1114E-06	3.589400E-06	-5.079300E-06
0.15	0.000003080	-1.44628E-05	7.18550E-06	1.01040E-05	-5.4302E-06	-6.276000E-06	2.294100E-06
0.20	0.000004154	-3.36426E-05	8.78800E-06	4.93150E-06	-1.80657E-05	-1.652190E-05	1.140900E-06
0.25	0.000005458	-3.69304E-05	7.21630E-06	-2.38800E-06	-2.94385E-05	-2.482420E-05	5.316430E-05
0.30	0.000006975	-3.47217E-05	4.33000E-08	-1.58484E-05	-3.54342E-05	-3.387290E-05	5.679730E-05
0.35	0.000008563	-3.59627E-05	-3.21180E-06	-2.40838E-05	-4.21559E-05	-4.537870E-05	5.832740E-05
0.40	0.000010124	-0.000044122	9.42570E-06	-3.09010E-05	-5.56749E-05	-3.222500E-05	5.304810E-05
0.45	0.000011702	-6.85257E-05	3.64856E-05	-5.13554E-05	0.000129912	1.636133E-04	2.944420E-05
0.50	0.000013265	-0.000131248	7.62651E-05	-9.44847E-05	0.000171055	1.439191E-04	-3.068900E-05
0.55	0.000013163	-0.00025817	1.21350E-04	-1.70607E-04	0.000198202	3.988430E-05	-1.326537E-04
0.60	0.000011918	-0.000398736	1.94862E-08	-3.04878E-04	0.000240536	-8.992800E-05	-2.095889E-04
0.65	0.000030656	-0.000352332	-1.54475E-04	-2.25555E-04	0.000364524	-1.141161E-04	-1.465103E-04
0.70	0.000072718	-8.03945E-05	-9.32032E-05	6.97866E-05		-3.597930E-05	3.678540E-05
0.75	0.000094029	0.000167108	5.21223E-05	3.05934E-04		4.825170E-05	1.972204E-04
0.80	0.000079077	0.000277176	1.56273E-04	3.77418E-04	0.00040638	6.703480E-05	2.640921E-04

Table S21: Imaginary part of the energies of the ground and excited states of $B(OH)_3$, at the CC3 level of theory, plotted in figs. 8-9.

B	$^1A'$	$^1E'_1$	$^1E'_2$	$^1A''$	$^1E''_1$	$^1E''_2$	
0.00	0.00000000	0.000000000	4.66578E-05	-4.66578E-05	0.000000000	5.13331E-05	-0.0000513331
0.05	-1.5163E-06	-2.91564E-05	5.62757E-05	9.4869E-06	5.62188E-05	4.88148E-05	-0.0000384837
0.10	-3.1322E-06	0.000030696	0.000094154	7.97992E-05	6.24372E-05	4.46173E-05	0.0000015281
0.15	-4.8396E-06	9.87508E-05	0.000136967	0.000157409	5.42475E-05	3.85138E-05	0.0000659032
0.20	-6.6378E-06	0.00010224	0.000167311	0.000213847	3.02004E-05	3.85667E-05	0.0001122227
0.25	-8.7336E-06	8.08258E-05	0.00020155	0.000241509	2.09522E-05	5.57919E-05	-0.0001357661
0.30	-1.11985E-05	8.15121E-05	0.000221318	0.000230545	4.11463E-05	8.21586E-05	-0.0001288078
0.35	-1.35377E-05	0.00011438	0.000186409	0.000184083	8.69196E-05	0.00010527	-0.0001123305
0.40	-1.44992E-05	0.000189684	0.000117769	0.000145262	0.000150204	-0.000317763	-0.0000716209
0.45	-1.29748E-05	0.000324817	0.000028205	0.000111896	-0.000559263	-0.000298814	0.0000276377
0.50	-1.14462E-05	0.000558309	-9.03826E-05	9.32414E-05	-0.000698702	-0.00014734	0.0002105807
0.55	-2.14757E-05	0.000903035	-0.000238055	9.63503E-05	-0.000863438	0.000139307	0.0004260225
0.60	-7.22035E-05	0.001154188	-0.000263603	8.70923E-05	-0.001156961	0.000409512	0.0004545325
0.65	-0.000195793	0.000855243	0.000379132	-0.000292816	-0.001555215	0.000323701	0.0000831134
0.70	-0.000322559	6.29959E-05	0.000350607	-0.001001833	-0.001812207	-0.000163457	-0.0004916238
0.75	-0.000325379	-0.000591891	-6.86969E-05	-0.001366638	-0.001691472	-0.000608465	-0.0008612312
0.80	-0.000223767	-0.000838728	-0.000415553	-0.001213615	-0.001309341	-0.000705062	-0.0009120412

Table S22: Imaginary part of the energies of the ground and excited states of $B(OH)_3$, at the CCSD level of theory, plotted in figs. 8-9.
